# Picosecond supercontinuum generation in large mode area photonic crystal fibers for coherent anti-Stokes Raman scattering microspectroscopy

**DOI:** 10.1038/s41598-018-27811-5

**Published:** 2018-06-22

**Authors:** Yujie Shen, Alexander A. Voronin, Aleksei M. Zheltikov, Sean P. O’Connor, Vladislav V. Yakovlev, Alexei V. Sokolov, Marlan O. Scully

**Affiliations:** 1 Texas A& M University, College Station, TX 77843 USA; 2 Russian Quantum Center, ul. Novaya 100, Skolkovo, Moscow Region, 143025 Russia; 3 Physics Department, International Laser Center, M. V. Lomonosov Moscow State University, Moscow, 119992 Russia; 4 Baylor University, Waco, TX 76798 USA; 5 Kazan Quantum Center, A.N. Tupolev Kazan National Research Technical University, Kazan, 420126 Russia

## Abstract

We perform a detailed theoretical and experimental investigation of supercontinuum generation in large-mode-area photonic crystal fibers pumped by a high-energy, high-repetition rate picosecond Nd:YVO_4_ laser, with the goal of using it as the Stokes beam in coherent anti-Stokes Raman scattering setup. We analyze the influence of fiber structure and length on the supercontinuum power, spectral shape, and group delay dispersion. We identify the experimental conditions for stable supercontinuum generation, with microjoule-level pulse energy and the spectrum extending beyond 1600 nm, which allows excitation of Raman frequencies up to 3000 cm^−1^ and beyond. We demonstrate reliable and efficient operation of a coherent anti-Stokes Raman spectroscopy and microscopy setup using this supercontinuum source.

## Introduction

**Figure 1:**
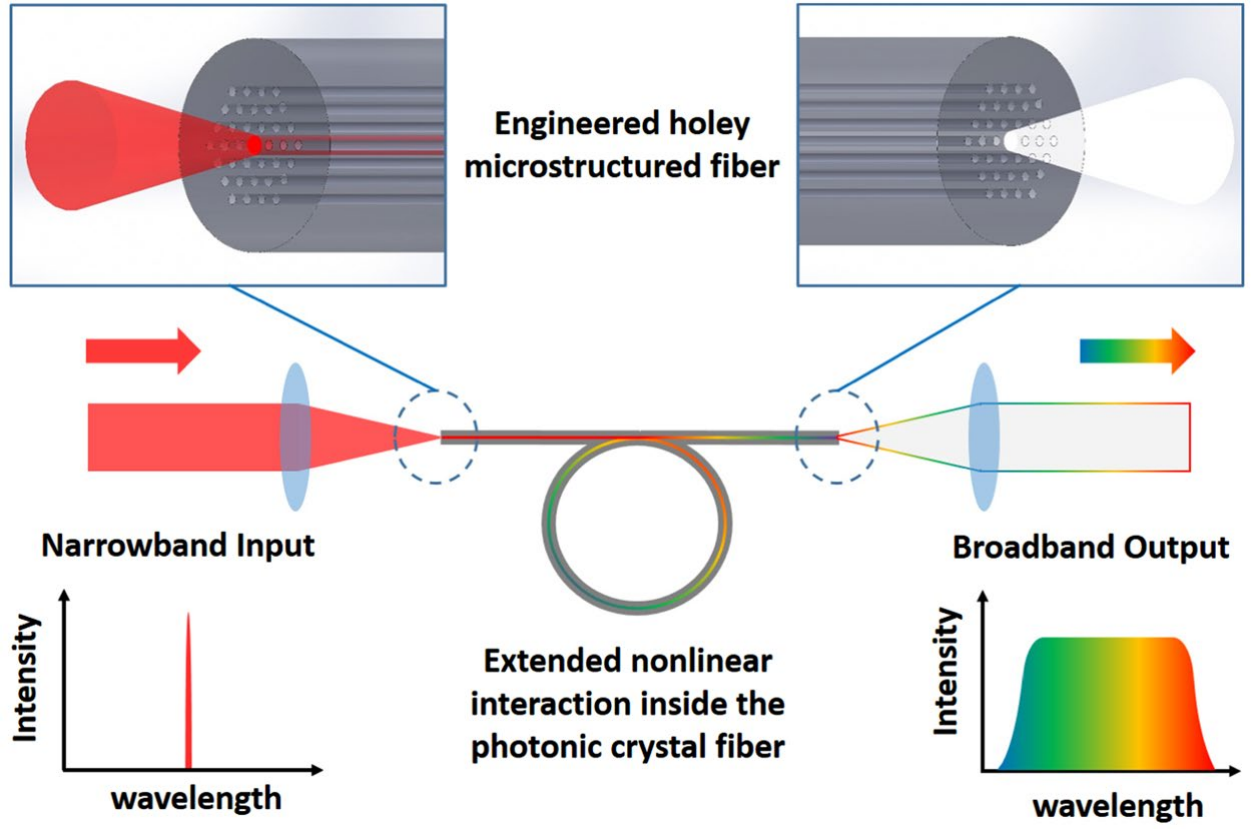
Supercontinuum generation in photonic crystal fiber. Specially engineered holey structure allows controlled dispersion in the fiber, which in turn assists various nonlinear interactions such as stimulated Raman scattering, parametric four-wave mixing, soliton evolution and dispersive wave generation, leading to significant spectral broadening.

The past decades have seen significant development in supercontinuum (SC) generation. In particular, the field has surged since the introduction of photonic crystal fiber (PCF). PCF-based SC generation (depicted in Fig. 1) has attracted substantial research interest^1^, and has led to novel applications in diverse fields such as optical communication, metrology, medical diagnostics and biophotonics^2–6^. Particular techniques that have seen important advances include optical coherence tomography and coherent anti-Stokes Raman scattering (CARS) microspectroscopy^1,7^. CARS is a nonlinear optical process; it has been shown to produce stronger signal as compared to spontaneous Raman scattering, and is free from the single-photon fluorescence background. CARS is therefore considered to be a promising candidate for high-speed, non-invasive, and chemically selective imaging tool^8–11^. Incorporation of SC source can be found in early experiments using the multiplex CARS scheme^12–15^. Later on, SC generation was also adopted in other CARS schemes, such as single-beam^16^ and spectral focusing^17^ CARS. To increase the system robustness, all-fiber source CARS systems were implemented, where SC served as a key ingredient^18,19^. SC generation has also been applied to stimulated Raman scattering microscopy^20^. All these improvements have advanced the commercialization and practical application of coherent Raman scattering microspectroscopy^11^.

A widely adopted SC generation scheme, commonly used in CARS, involves a femtosecond Ti:sapphire laser pumping a short (several centimeters) small-core high-nonlinearity PCF^7,16,17^. Other excitation sources such as sub-nanosecond microchip laser^15^ or Er-doped fiber lasers^21^ have also been implemented. On the other hand, large mode area (LMA) PCFs with core sizes above 10 *μ*m, which commonly possess low nonlinearity and are therefore suitable for light delivery^22,23^, have been found applicable for SC generation. Early research had analyzed SC spectra obtained using different LMA PCFs pumped by a nanosecond laser^24^, but with relatively low pump power (~100 mW) and long fiber length (100 m). Others had used amplified femtosecond laser to excite short-length LMA PCF^25,26^. While achieving *μ*J-level pulse energy with an octave-spanning spectrum, that scheme had only produced an average power on the level of mW. A picosecond pump source was also implemented, in an experiment focused on LMA PCF with large air-filling ratio^27^. For more common, commercially available LMA PCF, one expects to achieve *μ*J-level pulse energy, and simultaneously reaching Watt-level output power with an appropriate repetition rate of the pump source^28^. However, more careful characterization of such SC pulse is required, and systematic study of its dependence on various fiber parameters is needed in order to optimize the design for a CARS system. Such characterization will also serve as a useful reference for other applications of SC generation in LMA PCF.

In terms of the CARS application, there have been a few demonstrations using SC generation in LMA fibers^28,29^, and our present work builds upon these proof-of-principle experiments. The LMA-PCF-based SC source offers several advantages. The large core size can sustain significantly more power as compared to small-core, highly nonlinear PCF, offering bright SC output with both high pulse energy and high average power–this is beneficial for CARS as the increased pulse energy enhances signal level^30^. And it should be noted that SC power scaling is not straightforward: for a given pulse duration, pump power is narrowly constrained by the fiber used, and in order to, for example, scale up the generated SC power, the whole experiment, including the fiber, needs to be redesigned. Another advantage is that the large core sizes of these fibers allow less complicated optics and easier alignment for light in-coupling, as compared to small-core fibers; and the LMA fibers are easier to process in terms of splicing and connectorization, allowing implementation in an all-fiber setup^24^. When pumped with a picosecond laser slightly above 1000 nm, as we will show in this manuscript, the generated SC spectrum is predominantly red-shifted, and shows good flatness and smoothness in the infrared region–which forms an ideal combination for multiplex CARS, providing good spectral resolution, broad coverage of Raman bands, and minimal damage to cells and tissue^31,32^. Such a source is very promising for potential CARS applications in microscopy^33^, cell cytometry^28^, as well as in combustion diagnostics^34^.

In this report, we investigate the SC generation in several commercially available LMA PCF. We show that high power SC can be efficiently generated with spectrum extending beyond 3000 cm^−1^ from the excitation wavelength. Spectral and temporal behavior of the SC from different fibers are characterized in details, and we evaluate how different fiber parameters affect the SC generation process. Computer simulations are carried out to provide insights into the processes underlying the SC generation. As a final result, we demonstrate CARS spectroscopy and microscopy utilizing this novel SC source, and show improved spectral coverage compared to previous works^28,29^. A list of abbreviations used in the paper has been given in Table 1 for easy reference.

**Table 1:** Abbreviations used in the main text.

Acronym	Full Expression
SC	supercontinuum
LMA	large mode area
PCF	photonic crystal fiber
CARS	coherent anti-Stokes Raman scattering
FWM	four-wave mixing
ZDW	zero dispersion wavelength
GVD	group velocity dispersion
PS	polystyrene
PMMA	poly(methyl methacrylate)

## Results

### Theory and simulations

To understand the mechanisms of the SC generation process in LMA PCF, we first performed theoretical simulations in a 10-m-long LMA-20 fiber, and visualize the SC development with increasing excitation powers. Our numerical analysis was based on the generalized nonlinear Schrödinger equation^2,35–37^ for the field envelope:

1 $$\frac{\partial }{\partial z}A(\omega ,z)=i\tilde{D}(\omega )A(\omega ,z)-\frac{\alpha (\omega )}{2}A(\omega ,z)+\tilde{F}\{i\frac{{\omega }_{0}{n}_{2}\tilde{T}}{c}[{\int }_{-\infty }^{\infty }R(t-t^{\prime} )I(t^{\prime} ,z)dt^{\prime} ]A(t,z)\}\mathrm{.}$$

Here, *A*(*t*, *z*) is the field envelope, *A*(*ω*, *z*) is its Fourier transform, and *I*(*t*, *z*) = |*A*(*t*, *z*)|^2^. *z* is the coordinate along the propagation axis, *t* is the retarded time, *ω* = 2*πc*/*λ* is the radiation frequency, and *λ* is the wavelength. Fiber parameters used in the equation include: attenuation coefficient *α*(*ω*), propagation constant *β*(*ω*), and nonlinear refractive index *n*_2_. Other operators used in Eq. 1 include:

- $\tilde{F}$ is the Fourier transform operator.
- $\tilde{D}$ represents the dispersion terms, and is given by $\tilde{D}=\beta (\omega )-\beta ({\omega }_{0})-\partial \beta /\partial \omega {|}_{{\omega }_{0}}(\omega -{\omega }_{0})$, with center frequency *ω*_0_.
  - $$\tilde{T}=1+i{\omega }_{0}^{-1}\partial /\partial t$$.
  - *R*(*t*) is the optical response function, given by *R*(*t*) = (1 − *f*_*R*_)*δ*(*t*) + *f*_*R*_*h*(*t*). Each term is explained below.
  - (1 − *f*_*R*_)*δ*(*t*) represents the instantaneous (Kerr-type) response, and *δ*(*t*) is the delta function.
  - *f*_*R*_*h*(*t*) represents the retarded (Raman-type) response, in which *f*_*R*_ is the fraction of Raman nonlinearity in the nonlinear response. *h*(*t*) represents the Raman response function, and was approximated with a standard damped-oscillator model^2^, $h(t)=({\tau }_{1}^{2}+{\tau }_{2}^{2}){\tau }_{1}^{-1}{\tau }_{2}^{-2}\exp (-t/{\tau }_{2})\sin (t/{\tau }_{1})$.

**Figure 2:**
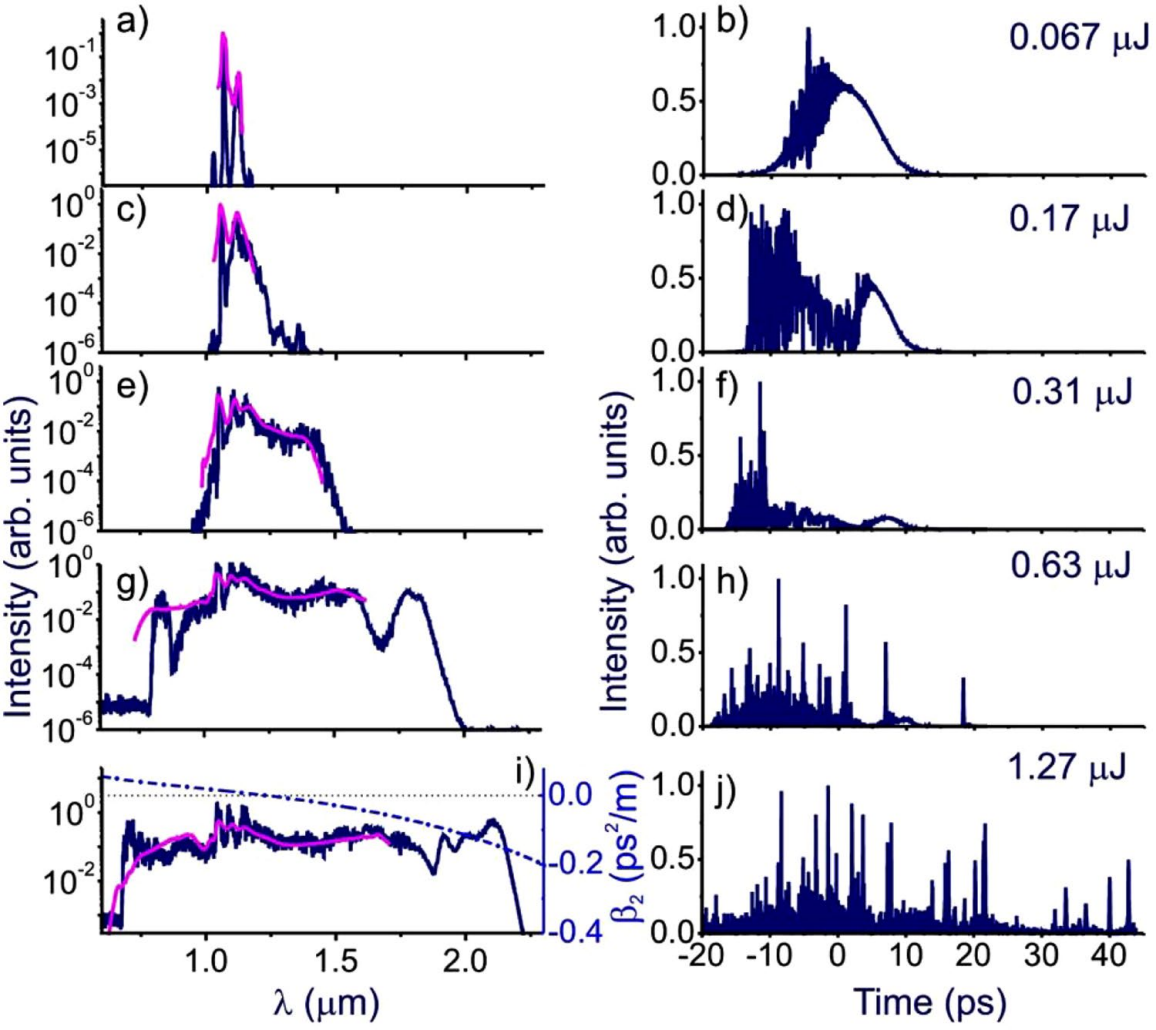
The SC output of the 10-m-long LMA-20 PCF: experiments (pink line) and simulations (blue line). The energy of the SC output is 0.067 *μ*J (**a**,**b**), 0.17 *μ*J (**c**,**d**), 0.31 *μ*J (**e**,**f**), 0.63 *μ*J (**g**,**h**), and 1.27 *μ*J (**i**,**j**). The fiber dispersion profile is shown by the dash-dotted line in (i).

Simulations were performed for typical parameters of fused silica^2,35^: *n*_2_ = 3.2 × 10^−16^ cm^2^/W, *f*_*R*_ = 0.18, *τ*_1_ = 12.5 fs, and *τ*_2_ = 34 fs. The fiber dispersion profile was defined in such a way as to mimic the dispersion of the LMA-20 fiber with the group-velocity dispersion (GVD) passing through the zero at the wavelength *λ*_*z*_ ≈ 1.23 *μ*m, as shown in Fig. 2(i). The nature of the physical problem, which deals with broadband SC field waveforms generated by narrowband, picosecond input laser pulses, propagating over large fiber lengths, dictates a high computation complexity of simulations^38^. For an adequate description of SC generation by picosecond laser pulses in our experiments, simulations have been performed on a computational grid consisting of 2^17^ nodes with a 0.5-fs step in the time variable and 2 × 10^5^ nodes with a 50-*μ*m step along the z-coordinate.

When performed on such a computation grid, simulations provided an accurate description of the SC output of the PCF we measured. Figure 2 displays the simulation results for the 10-m-long LMA fibers, revealing important tendencies in SC generation and providing important insights into the physics behind spectral and temporal transformations of picosecond laser pulses in LMA PCFs. As is clearly seen in Fig. 2(a) and (b), the initial stage of the SC generation is dominated by stimulated Raman scattering, in good agreement with prior experimental data^39^. Intense Raman sidebands are readily visible in the PCF output for input laser energies in the range of 70 nJ (Fig. 2(a)). As the input laser energy is increased to about 100 nJ, higher-order Raman sidebands start to show up in the fiber output. At even higher input laser energies, these Raman sidebands seed a parametric four-wave mixing (FWM) decay of the pump into Stokes and anti-Stokes FWM sidebands. This parametric conversion is especially efficient near the zero dispersion wavelength (ZDW) where FWM processes of this type are automatically phase-matched^40^. The long-wavelength part of the SC field that falls in the range of the anomalous GVD gives rise to multiple optical solitons, which undergo a continuous self-frequency shift, due to the Raman effect, as the pulse propagates along the fiber. These Raman-shifted solitons are clearly visible as intense peaks in the temporal envelope of the SC output in Fig. 2.

**Figure 3:**
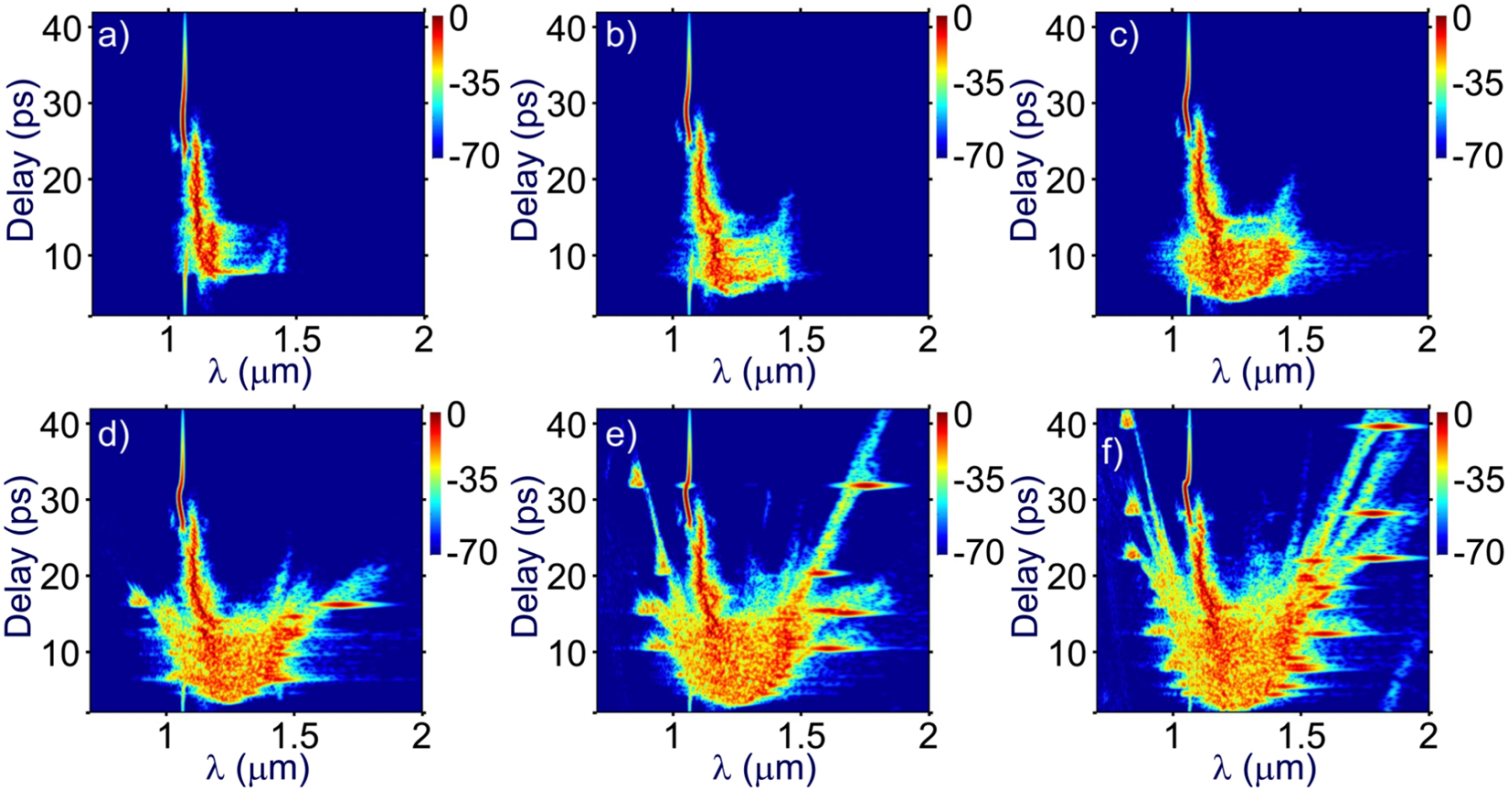
Spectrograms of the SC output from the 10-m-long LMA-20 PCF. The energies of the SC output are 0.24 *μ*J (**a**), 0.35 *μ*J (**b**), 0.41 *μ*J (**c**), 0.45 *μ*J (**d**), 0.56 *μ*J (**e**), and 0.63 *μ*J (**f**).

A clearer picture of the soliton development process is demonstrated in spectrograms shown in Fig. 3. Well-isolated features corresponding to the solitons are visible at the output when the SC energy reaches 0.41 *μ*J in Fig. 3(d), and increases in number as the pulse energy increases. Meanwhile, dispersive waves originated from these solitons also appear as isolated packets on the anti-Stokes side of the excitation wavelength, and their correlation can be directly drawn from the spectrograms. In addition, the effect of chromatic dispersion due to fibers are visualized in Fig. 3 as a parabolic variation of spectral components over wavelength.

To verify our theoretical model, we have collected SC spectra from the 10-m-long LMA-20 PCF with the corresponding pulse energy, and the results are plotted together with the theoretical simulation curves in Fig. 2. In general, the experimental spectra fall in good agreement with the simulation predictions in spectral shape and major sideband positions. Deviations in the peak structures could be the result of calibration errors, finite resolution, and limited dynamic range of the spectrometer module. Bending and tension in the fiber also contributes to the difference between the theoretical and the experimental data, as their effects are hard to quantify numerically for the simulation. We note that due to the limited detection range of the spectrometer we used, the measured SC spectrum is shown only up to 1700 nm, and future studies of the SC in the longer wavelength range requires more careful experimental design.

### SC generation with different fiber parameters

**Figure 4:**
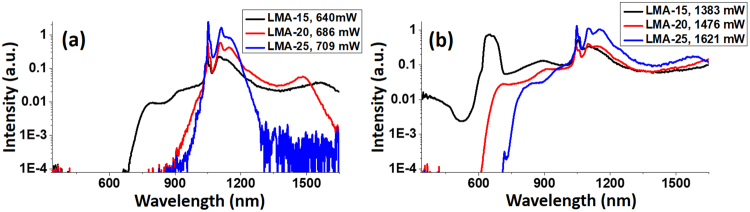
SC spectra generated from 5-m-long LMA-15, LMA-20, and LMA-25 fibers, under the pump power of (**a**) 1.36 W and (**b**) 3.8 W. Legends show the corresponding length and the SC power for each curve.

Given the fact that the LMA fibers studied here share similar dispersion profile and same core material, it is expected that the LMA-15 and LMA-25 PCF follow similar SC generation process as demonstrated in Fig. 2. However, the difference in core size and bent edge can lead to significant difference in the SC pulse energy and spectrum, especially on the short-wavelength side of the SC. We examine such differences by comparing the SC spectra generated from 5-m-long LMA-15, LMA-20, and LMA-25 fibers, as shown in Fig. 4. Figure 4(a) indicates that, under a relatively low pump power (1.36 W), SC spectrum in fiber with smaller core gained broader extension in wavelength compared to that with a larger core under the same excitation power. This is reasonable due to the smaller effective area, which leads to higher nonlinear coefficient and therefore enhanced wavelength extension. Under a higher pump power of 3.8 W, the SC spectra from the three fibers are shown in Fig. 4(b). Despite the similar broadening on the long-wavelength side, the SC spectra exhibited vast difference in the visible range, with the LMA-15 spectrum extended into the blue light region, the LMA-20 spectrum ended close to 600 nm, and the LMA-25 spectrum edge further red-shifted to 700 nm. Also, for the LMA-15 spectrum, we noticed a peak rising slightly above 600 nm. This could be attributed to the FWM process, in which the pump beam seeds distant spectral components generation that appear symmetrically on both sides of the excitation wavelength, and satisfy the phase-matching condition^35^. Here the Stokes-side peak was missing because it was out of the detection range, and further verification would require appropriate detector and experimental design.

We note that, while 3.8 W of pump power is close to the damage threshold of the LMA-15 fiber, for LMA-20 and LMA-25 the pump power can be safely increased up to 5 W and 8 W, respectively. But the corresponding SC power only rose to 1.6 W and 1.9 W, respectively, according to our measurement; and the spectra obtained from these fibers under higher pump power showed insignificant changes in their visible range performance. The major reason leading to such differences between fibers can be the excessive loss of light close to the bend edge of the fiber. Other factors such as changes in nonlinearity and ZDW can also affect the short-wavelength-side performance of the SC^24^.

**Figure 5:**
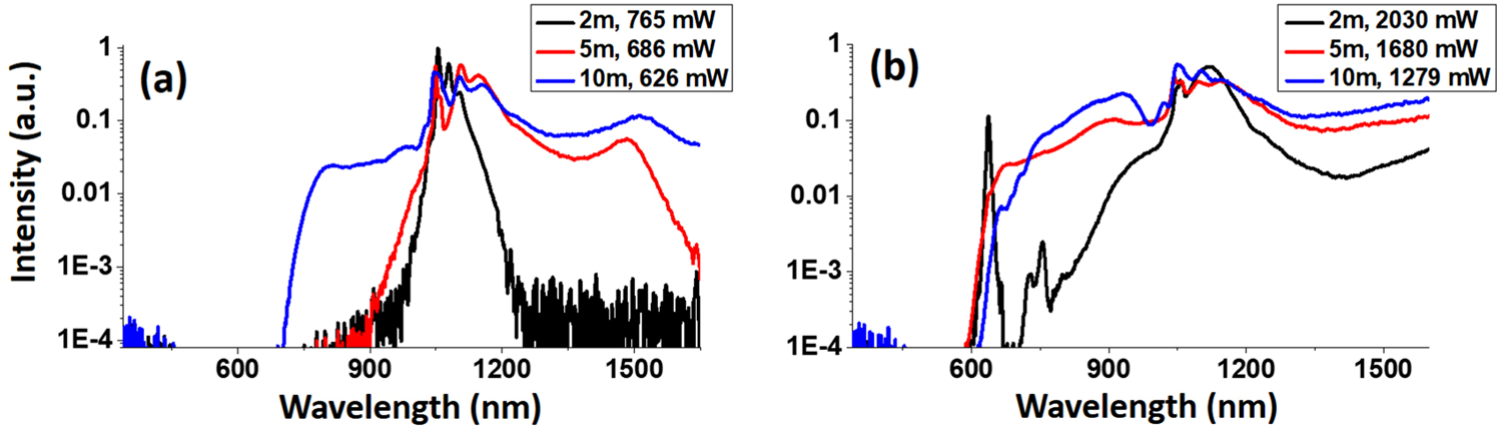
SC spectra generated from LMA-20 fiber with 2-meter, 5-meter, and 10-meter length, under a pump power of (**a**) 1.36 W and (**b**) 5.8 W. Legends show the length and output SC power of the corresponding curves.

Another factor influencing the output SC pulse is the fiber length. We expect longer fibers to have a lower onset power level for the spectral broadening beyond ZDW to occur, because the soliton fission length is inversely proportional to the square root of the peak intensity^35^. This is indeed the case, as we can see in Fig. 5(a) where three SC spectra obtained from the LMA-20 PCF with 2-meter, 5-meter, and 10-meter fiber length are plotted, all pumped at 1.36 W. While for the 2-m-long LMA-20, the SC spectrum extended only up to 1200 nm, those of 5-m-long and 10-m-long fibers had exceeded 1600 nm, and the SC generated in the 10-m-long fiber also showed significant extension on the short-wavelength side of the pump, corresponding to the simulation shown in Figs 2(g) and 3(f). The SC spectra under higher pump power (5.8 W) are shown in Fig. 5(b). Overall, we see that with longer fibers, the spectrum becomes flatter and smoother from the visible to the short-wave infrared region. For the 2-m-long LMA-20 fiber, the variation in spectral intensity exceeded 15 dB in the range from 1000 nm to 1600 nm, while that for 5-m-long and 10-m-long fibers were 7.7 dB and 6.5 dB, respectively. On the other hand, SC pulse energy saw significant decrease as the fiber length increase, which again could be attributed to the loss due to bending and tunneling modes.

**Figure 6:**
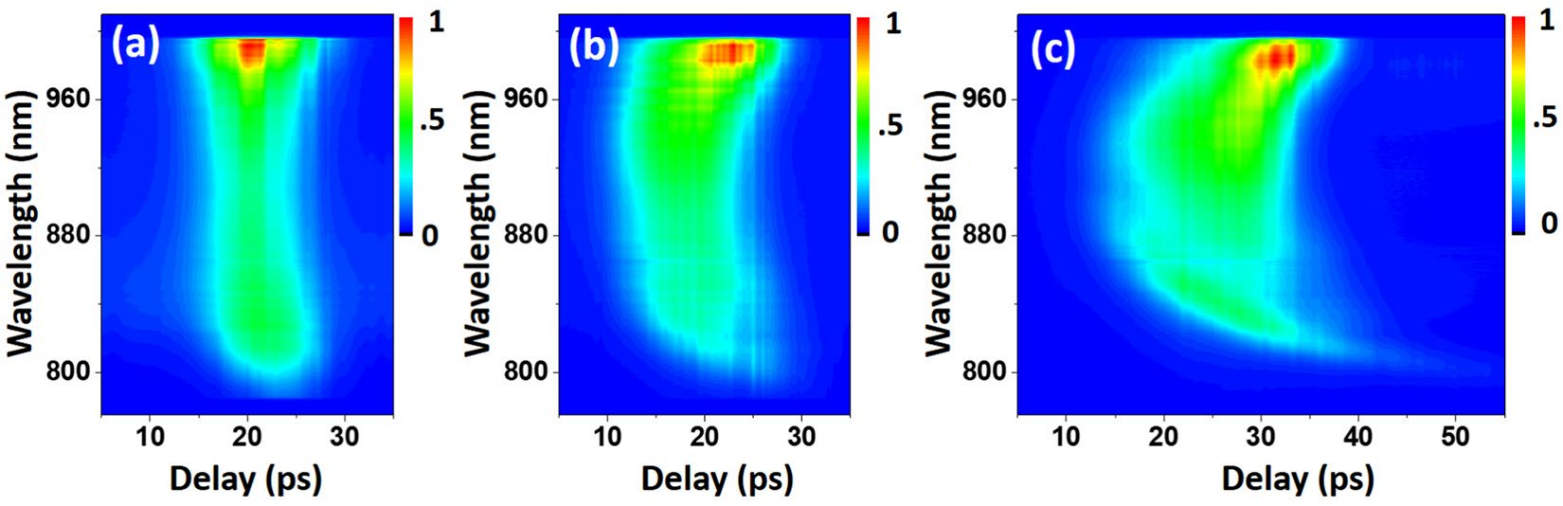
Spectrograms of the nonresonant anti-Stokes signal from a microscope slide. SC Stokes pulses are generated from LMA-20 fibers with a length of (**a**) 2 m, (**b**) 5 m, and (**c**) 10 m.

When varying fiber length, chromatic dispersion also plays an important role in SC characteristics, as we have already seen in the simulation result. To visualize and quantify the effect of fiber dispersion, we used the anti-Stokes nonresonant FWM signal from a microscope slide (Micro Slides, VWR, Inc.) to infer the temporal behavior of the generated SC. We measured a sequence of spectra at varying pump and Stokes delay times, and Fig. 6 shows three such spectrograms recorded for the SC generated in 2-m-, 5-m-, and 10-m-long LMA20 fibers. We notice the group delay dispersion and pulse-broadening effects become significant in the case of a longer fiber. For the 10-m-long LMA-20 fiber, the leading edge of the anti-Stokes signal pulse was around 935 nm, corresponding to ~1230 nm in the SC pulse, which is the ZDW of the fiber; and on either side of ZDW the pulse was stretched in time up to 25 ~30 ps. The spectrogram for the 2-m-long LMA-20 fiber, on the other hand, shows little broadening due to dispersion. The measured spectrogram in Fig. 6(c) exhibited similar chromatic dispersion property as that in Fig. 3(f), yet the fine details of the temporal behavior of the generated SC, such as those related to soliton mechanism, are not visible. This is because the pump pulse extends up to 7 ps in time, therefore lacks temporal resolution, and the peak information from various solitons tend to be smoothed out due to the convolution with the pump pulse. Either probing with a synchronized femtosecond laser or examining the SC pulse through optical time-stretch^41^ can be used to visualize the soliton behavior shown in Fig. 3(f).

**Figure 7:**
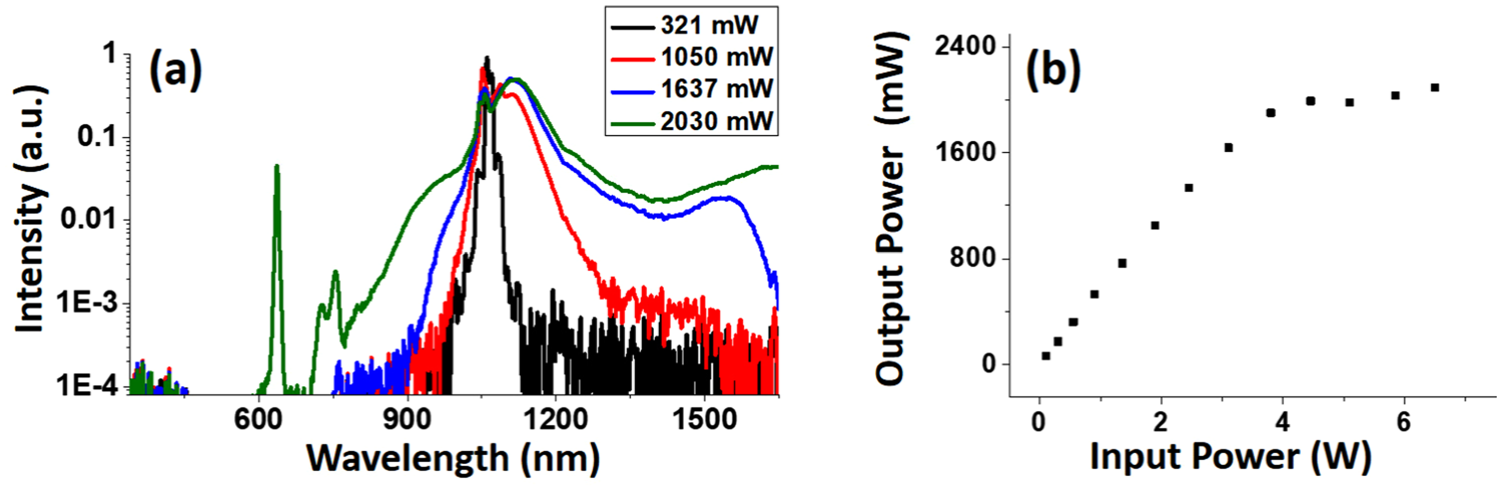
(**a**) SC spectra generated from a 2-m-long LMA-20 fiber. Legends show the output SC power of each curve. (**b**) The dependence of the SC output power as a function of the input power.

To evaluate the effect of bending loss associated with these fibers, we first take another look at how the SC develops with increasing pump power. Figure 7(a) shows a set of SC spectrum collected for a 2-m-long LMA-20 fiber under increasing excitation power, and the results followed a similar trend as that shown in the simulation in Fig. 2. We also plot the relationship between the incident power and the output SC power in Fig. 7(b). When the pump power was low, an increase in the pump power led to a linear growth of the SC power, and the throughput remained above 50%; when the excitation power exceeded 4 W, the growth rate of the SC power decreased sharply, and the SC power saw a saturation around 2 W. The turning point coincided with that when the SC started to develop its visible portion. It is reasonable to believe that the saturation is mainly due to the light loss close to the bend edge–the generated visible light leaks the fiber via higher-order modes or tunneling modes. Therefore, the excessive power used to generate this visible portion is lost during transmission, and the SC sees saturation in power.

**Figure 8:**
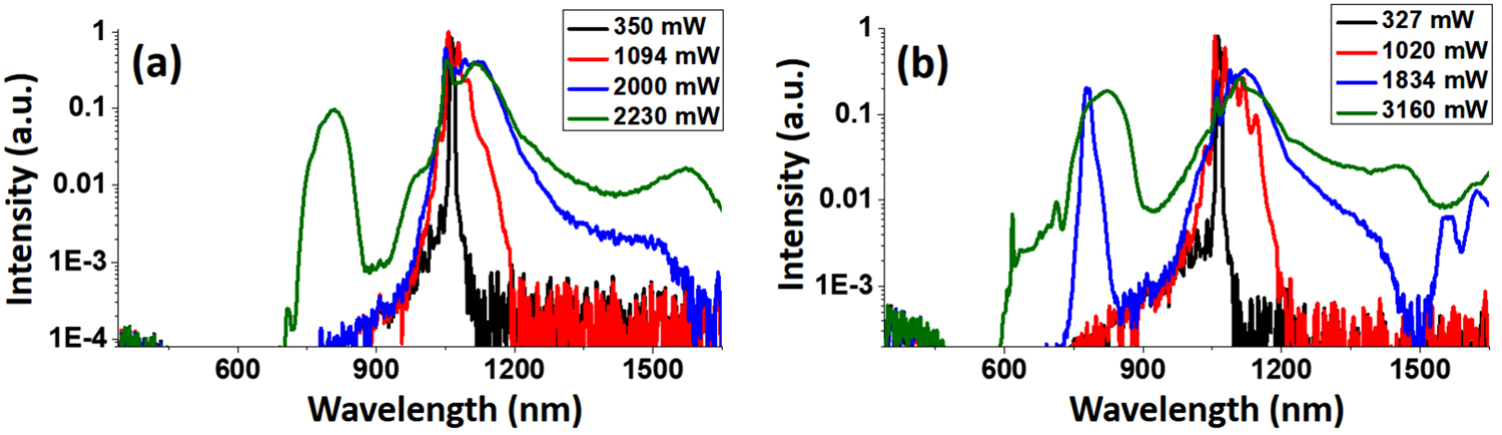
SC spectra generated from 2-m-long LMA-25 fiber in (**a**) bent and (**b**) straight status. Legends show the output SC power of each curve. The curves in the two plots with the same color shares the same excitation power.

For a direct comparison, we recorded the SC spectra from the 2-m-long LMA-25 fiber in bent and straight status. When bent in spool, the bending radius of the fiber was around 12.5 cm. The SC spectra from bent and straight fibers are plotted in Figs 8(a) and (b), respectively. As expected, the SC produced by the straight fiber displayed a major difference in the visible range performance, with the visible cut-off edge shifted from 700 nm to 600 nm; and the SC power increased from 2.2 W to 3.2 W under the same pump power of 8.3 W. This can be attributed to the reduced bending loss in the straight fiber, which preserved more power in the SC generation and transmission. Also, we notice that for the straight fiber spectrum, a distinct peak signal around 800 nm appeared when the SC power increased to 1.8 W. This anti-Stokes spectral component could also be due to FWM process, as we can see a corresponding Stokes component arose around 1590 nm which was otherwise absent in bent fiber. We note that such difference in SC spectrum under different fiber status could contribute to the deviation of theoretical and experimental results on the short-wavelength side shown in Fig 2(g) and (i).

**Figure 9:**
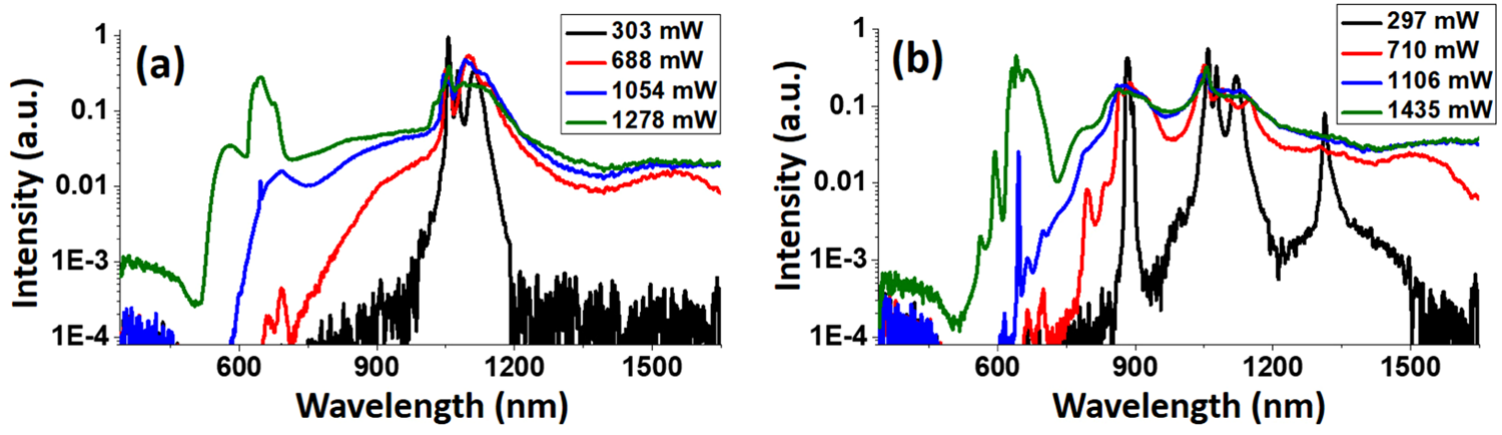
SC generation from the 2-m-long LMA-PM-15 fiber, with (**a**) and (**b**) showing excitation along the fast- and slow-axis, respectively. Legends show the output SC power of each curve.

Finally, we note that the SC produced in the previous fibers were not polarization-sensitive–that equal amount of energies are distributed in perpendicular polarizations. This was verified by monitoring the SC power and spectrum after a polarizer, and seeing that no significant change occurred when rotating the polarizer. In order to obtain efficient SC source for polarization-sensitive application, we tested the SC generation in the polarization-maintaining LMA fiber as well. A temporary microscope setup equipped with a 20× objective was used to image the input fiber end facet to determine the direction of the fast- and slow-axis of the fiber. The SC spectra from a 2-m-long LMA-PM-15 fiber are shown in Fig. 9, in which Fig. 9(a) corresponds to the case where the excitation polarization was along the fast-axis, and Fig. 9(b) corresponds to that along the slow-axis of the fiber. For the latter case, two spectral peaks emerged when SC power reached 710 mW, one around 890 nm and the other around 1320 nm. By passing the SC output through a polarizer, we found that these spectral components had a polarization perpendicular to the rest of the pulse. Again, this can be attributed to the FWM process^35^, only that the pump beam was seeding the mode with perpendicular polarization, and the peaks at 890 nm and 1320 nm correspond to the anti-Stokes and Stokes components, respectively. We note that the SC generation shown in Fig. 9(a) has a similar spectrum to that generated in the 2-m-long LMA-15 fiber, with a slightly lower output power of 1.3 W compared to the 1.6 W from the latter. However, given the fact that the output light was linearly polarized, the SC generation in the polarization-maintaining LMA fiber actually contained more useful power as compared to a typical LMA fiber, especially for polarization CARS application. Therefore it is reasonable to expect a polarization-maintaining version of the LMA-20 and LMA-25 PCF to produce higher power linearly polarized SC light with higher efficiency.

### CARS microspectroscopy

**Figure 10:**
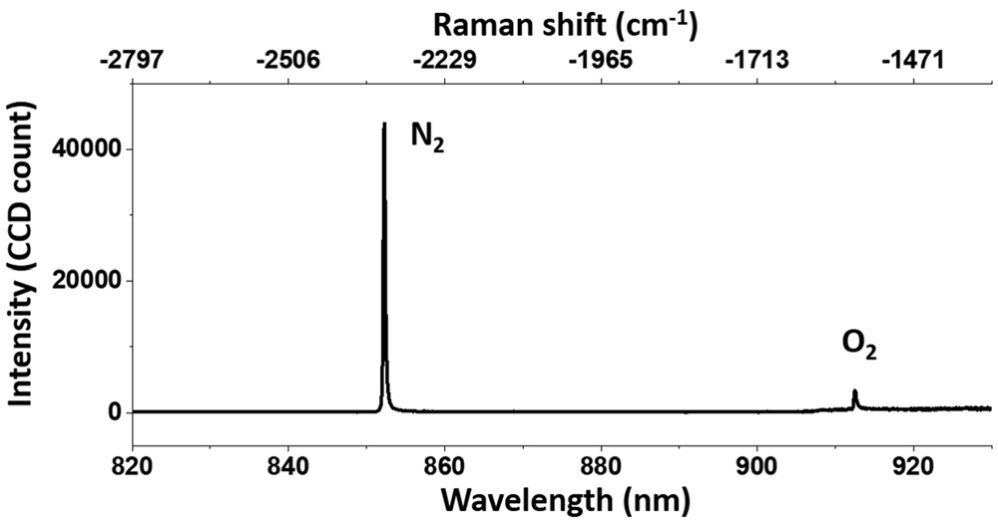
CARS signal collected from the ambient air using the picosecond SC setup, exhibiting two peaks corresponding to nitrogen (2331 cm ^−1^) and oxygen (1556 cm^−1^) vibrational transitions.

For demonstration of CARS microspectroscopy, we have chosen to use the 2-m-long LMA-20 fiber as the SC source. The short fiber length eliminated the need for large chirp compensation; and the LMA-20 PCF produced more useful Stokes power compared to the LMA-15 fiber, while requiring only moderate excitation power, leaving us plenty of room for power control in the pump beam. As an example, we first showed CARS spectroscopy of the ambient air. The power of the pump and Stokes beams were measured to be 89 mW and 139 mW, respectively. We loosely focused the beam into the air using a lens with the focal length of 100 mm (we estimated the beam-waist to be 30 *μ*m), and set the integration time to be 50 ms. Figure 10 shows a typical CARS spectrum obtained from the ambient air, with the vibrational peaks corresponding to nitrogen and oxygen molecules marked in the graph. The ratio of the nitrogen peak count over the oxygen peak count was around 15, which was reasonably close to the previous result^42^, taking into account the quadratic dependence of the CARS signal on the concentration and the variation in spectrometer efficiency.

**Figure 11:**
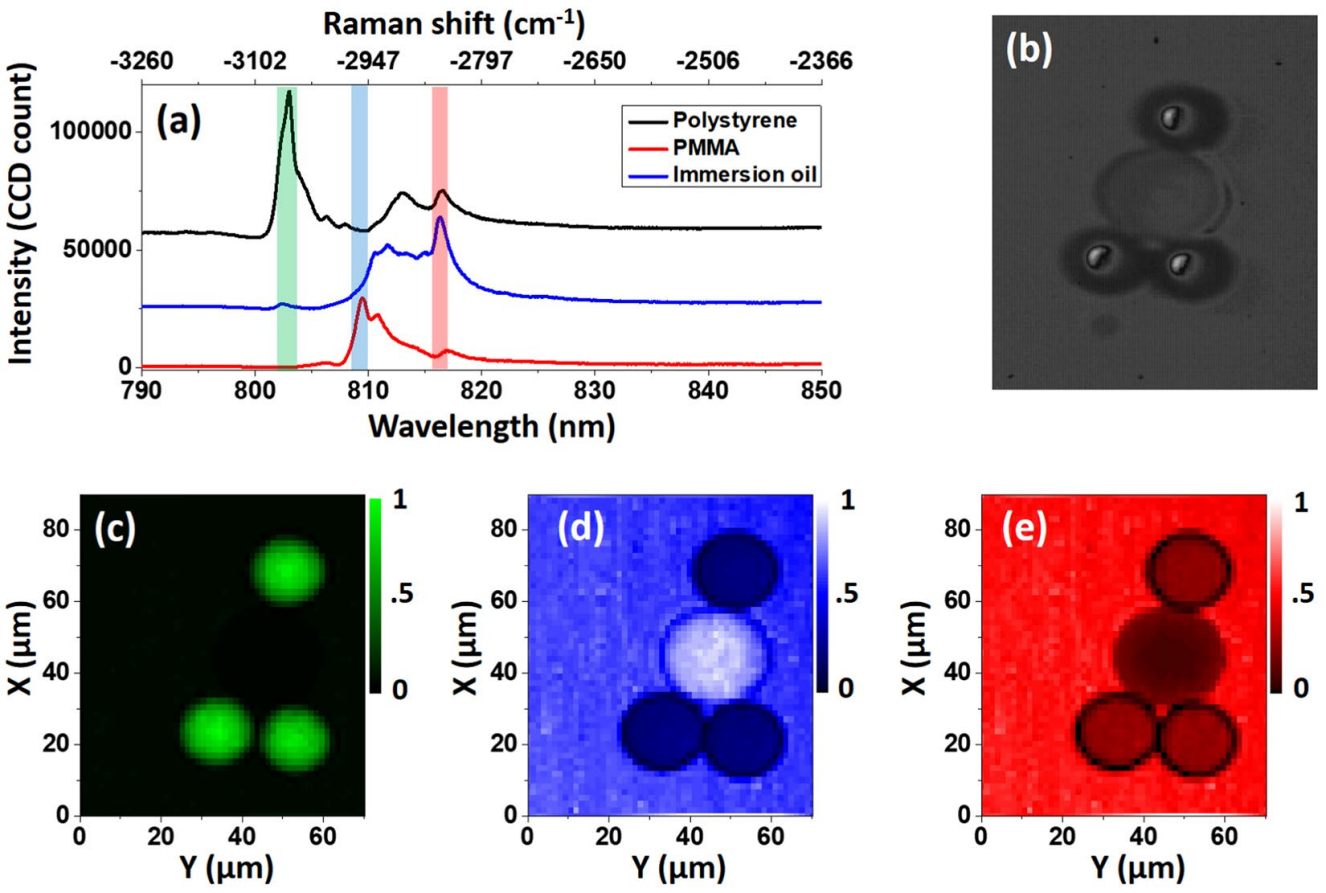
(**a**) CARS spectra from polystyrene, PMMA, and immersion oil. (**b**) Bright-field image of the captured bead structure. The retrieved image using the signal in the three bands marked in (**a**) are shown in (**c**), (**d**) and (**e**), plotted with their corresponding colors.

Next we performed CARS microspectroscopy on a mixed sample of polystyrene (PS) and poly(methyl methacrylate) (PMMA) beads, with bead size of 20 *μ*m (74491, SigmaAldrich, Inc.) and 30 *μ*m (77523, SigmaAldrich, Inc.), respectively. The beads were surrounded by an immersion oil (Type B, Cargille Lab), and the whole mixture was sandwiched between two coverslips (Micro Cover Glasses, VWR). Typical CARS spectra for those three species are shown in Fig. 11(a), and the bright-field image is shown in Fig. 11(b). We selected three characteristic bands in the C-H stretching region corresponding to each material for imaging. The center Raman shifts were chosen to be 3050 cm^−1^, 2950 cm ^−1^, 2850 cm ^−1^ for PS, PMMA and immersion oil, respectively. The retrieved images from these bands are shown in Fig. 11(c,d) and (e), respectively. From these figures we can easily identify the PS and PMMA beads from the background. The power of the pump and Stokes beams were 1.9 mW and 1.3 mW, respectively, with a pixel dwell time set to 40 ms. The focusing element was an off-axis parabolic mirror which had a focal length of 15 mm, and signal was collected by an achromatic lens with 30 mm focal length. We estimate the spatial resolution to be 4 *μ*m. We note that the selection of the dwell time was limited by the CCD shutter time, which has an open/close time around 10 ms. It is possible to reduce the exposure time by adding an additional fast shutter in the laser path to limit the exposure below 10 ms, which is more typical for multiplex CARS setup^10,30^. We also note that there is still much space for improvement on the signal level, due to the low N.A. (0.37) focusing element used here.

**Figure 12:**
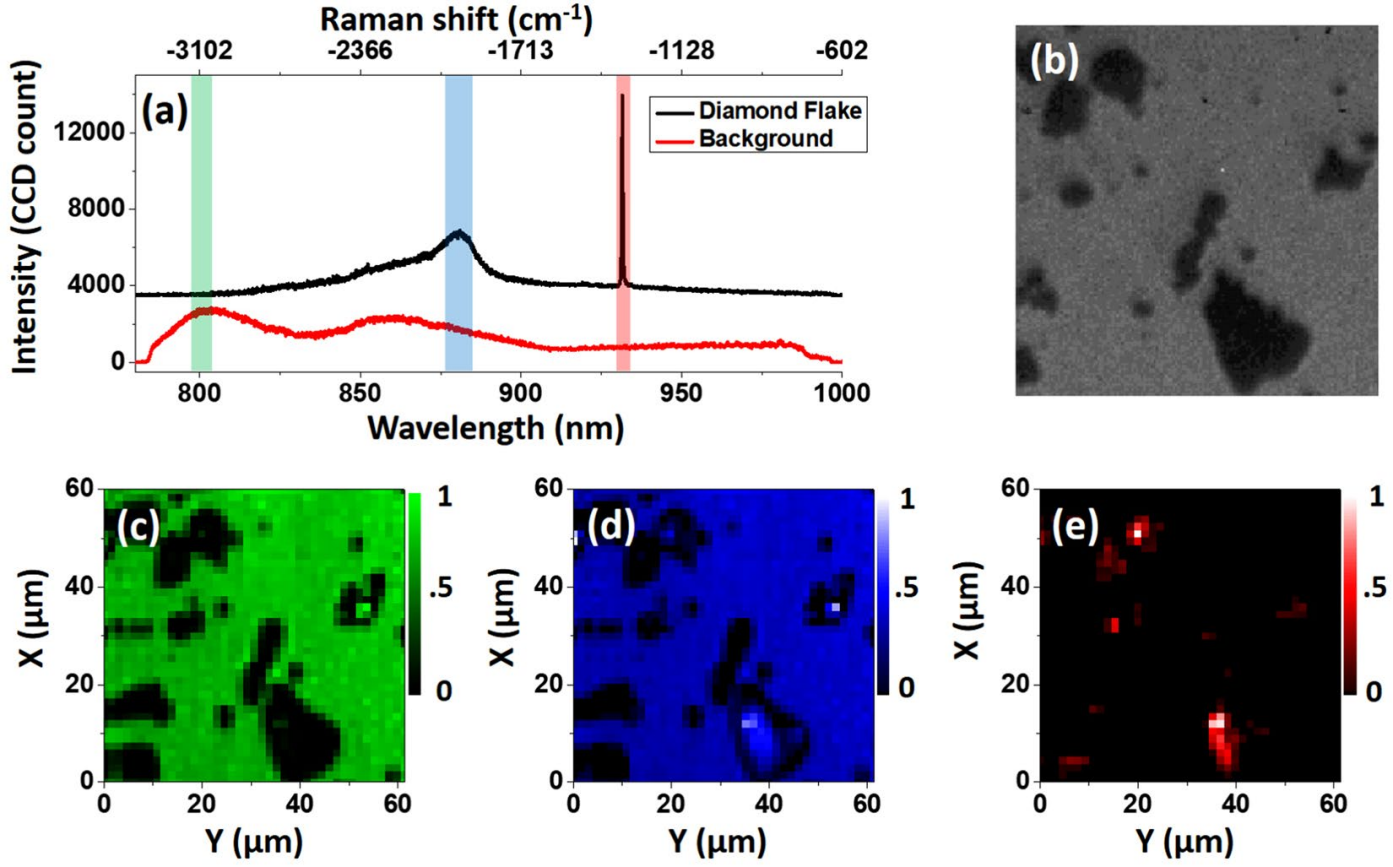
(**a**) CARS spectra from diamond flakes. (**b**) Bright-field image of the captured flake structure. The retrieved image using the signal in the three bands marked in (**a**) are shown in (**c**), (**d**) and (**e**), plotted with their corresponding colors.

We also demonstrated imaging of micron-size diamond flakes with nickel embedded in it^43^. A typical CARS spectrum of the diamond sample and the background signal is exhibited in Fig. 12(a). The diamond signal at 1332 cm ^−1^ is clearly pronounced, and the peak around 885 nm is due to nickel^43^. Again, a bright-field image of the structure captured is shown in Fig. 12(b), and the three retrieved CARS images corresponding to the background, nickel, and diamond are presented in Fig. 12(c,d) and (e), respectively. Here the power of the pump and Stokes beams were 5.1 mW and 3.9 mW, respectively, with the pixel dwell time 40 ms. We note that, due to the opacity of these flakes, the epi-detection CARS setup^11^ could yield even higher signal level compared to the signal detected in transmission geometry, as it was used here.

## Discussion and Conclusion

The typical coupling efficiencies for all fibers in a low power regime were around 60%, mostly due to input coupling loss. Higher coupling efficiency (up to 90%) could be achieved through spatially filtering the laser mode. However, this imposes a large waste on the laser power and heating load on the spatial filter. Further improvement can be made using wavefront correction tool.

CCD etaloning effect during spectral acquisition was found to be significant. Because the fingerprint region is between 900 and 1010 nm, for back-illuminated CCD, etaloning will produce serious fringing problems on spectrum, especially when the signal level is high and the nonresonant background is prominent. We came up with a simple solution to this challenge by utilizing a single-mode fiber for coupling into the spectrometer. If the fiber’s core-size is smaller than the CCD pixel size (about 15 *μ*m), then, by recording the spectrum of a calibrated source, say, a halogen lamp, a correction curve can be measured and the fringe on the spectrum can be suppressed through post-processing. In addition, the function of the single-mode fiber is similar to the pinhole in a confocal microscope, thus, offering additional advantage in enhancing spatial resolution in imaging.

Finally we note that, given the efficiency roll-off of the CCD close to and above 1000 nm, it was necessary to assign more power in the low wavenumber region of the Stokes pulse so that the final CARS signal had higher signal-to-noise ratio across the whole detection range. In this sense, it is reasonable to use a 2-m-long fiber, which offers optimal power ratio and produces relatively insignificant group delay dispersion.

In conclusion, we have demonstrated SC pulse generation in LMA PCF with microjoule-level pulse energy and Watt-level output power. Different fiber parameters have been tested, and their effects on the spectral and temporal behavior of the generated SC were evaluated. Theoretical simulation has been carried out to provide additional insights into mechanisms of the SC generation, and showed good agreement with the experimental results. We have demonstrated CARS spectroscopy in the ambient air, as well as CARS imaging of microspheres and diamond flakes using this optimized SC source. We believe this will be an alternative and preferable CARS source for biomedical and other applications^30^.

## Methods

**Figure 13:**
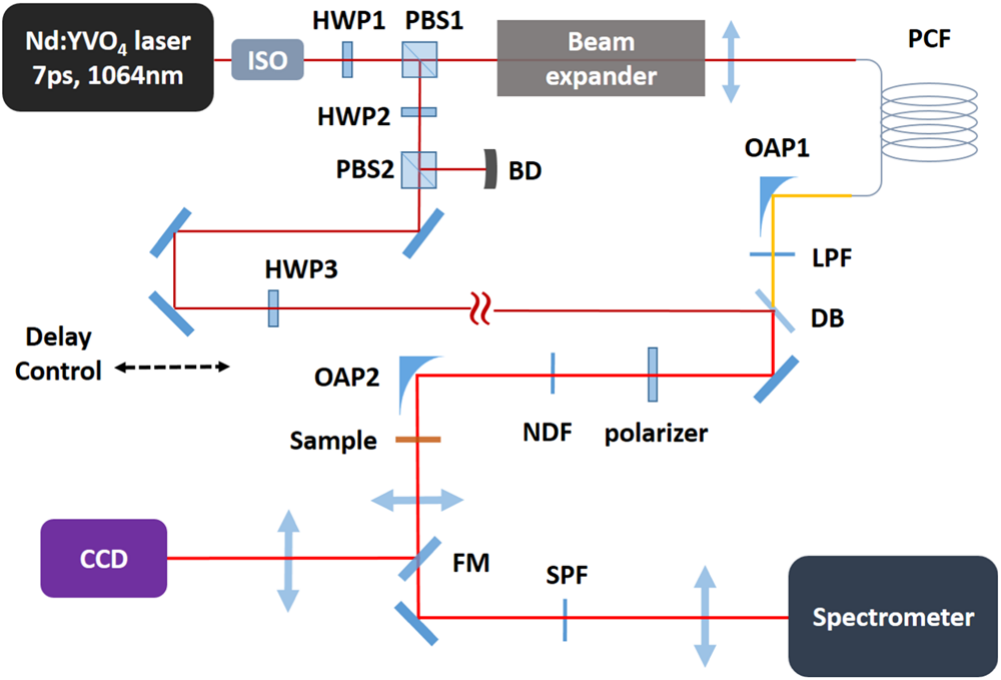
Schematic of the picosecond supercontinuum coherent anti-Stokes Raman scattering system. ISO: isolator; HWP: half-wave plate; PBS: polarizing beam-splitter; BD: beam dump; OAP: off-axis parabolic mirror; LPF: long-pass filter; DB: dichroic beam-splitter; NDF: neutral density filter; FM: flip mirror; SPF: short-pass filter.

The experimental setup is shown in Fig. 13. An industrial Nd:YVO_4_ laser centered at 1064 nm (APLX-10, Attodyne, Inc.) was used as the pump source, which output 7 ps pulses at 1-MHz repetition rate. The output beam was divided into two arms, and the power in each arm was controlled by the combination of a half-wave plate and a polarizer. The output of one arm was launched into the LMA PCF for the SC generation using a plano-convex lens (LA1509-C, Thorlabs, Inc.), and was subsequently collimated by an off-axis parabolic mirror (MPD129-P01, Thorlabs, Inc.); the output of the other arm was sent to a delay stage to ensure a proper temporal overlap at the sample.

The SC beam was filtered by a long-pass filter (FELH1150, Thorlabs, Inc.) before recombining collinearly with the 1064-nm beam through a dichroic beam-splitter (LPD02-1064RU-25, Semrock, Inc.). The SC was used as the Stokes beam, and the 1064 nm radiation was used as the pump beam in the CARS experiment. Both beams were aligned to the same polarization and attenuated before focusing into the sample. The transmitted light was either directed to a CCD camera by a flip mirror for bright-field imaging, or guided through a short-pass filter (FESH1000, Thorlabs, Inc.) and then sent to the spectrometer (Holospec, Andor, Inc) with the attached TE-cooled CCD (iDus416, Andor, Inc) for detection.

**Figure 14:**
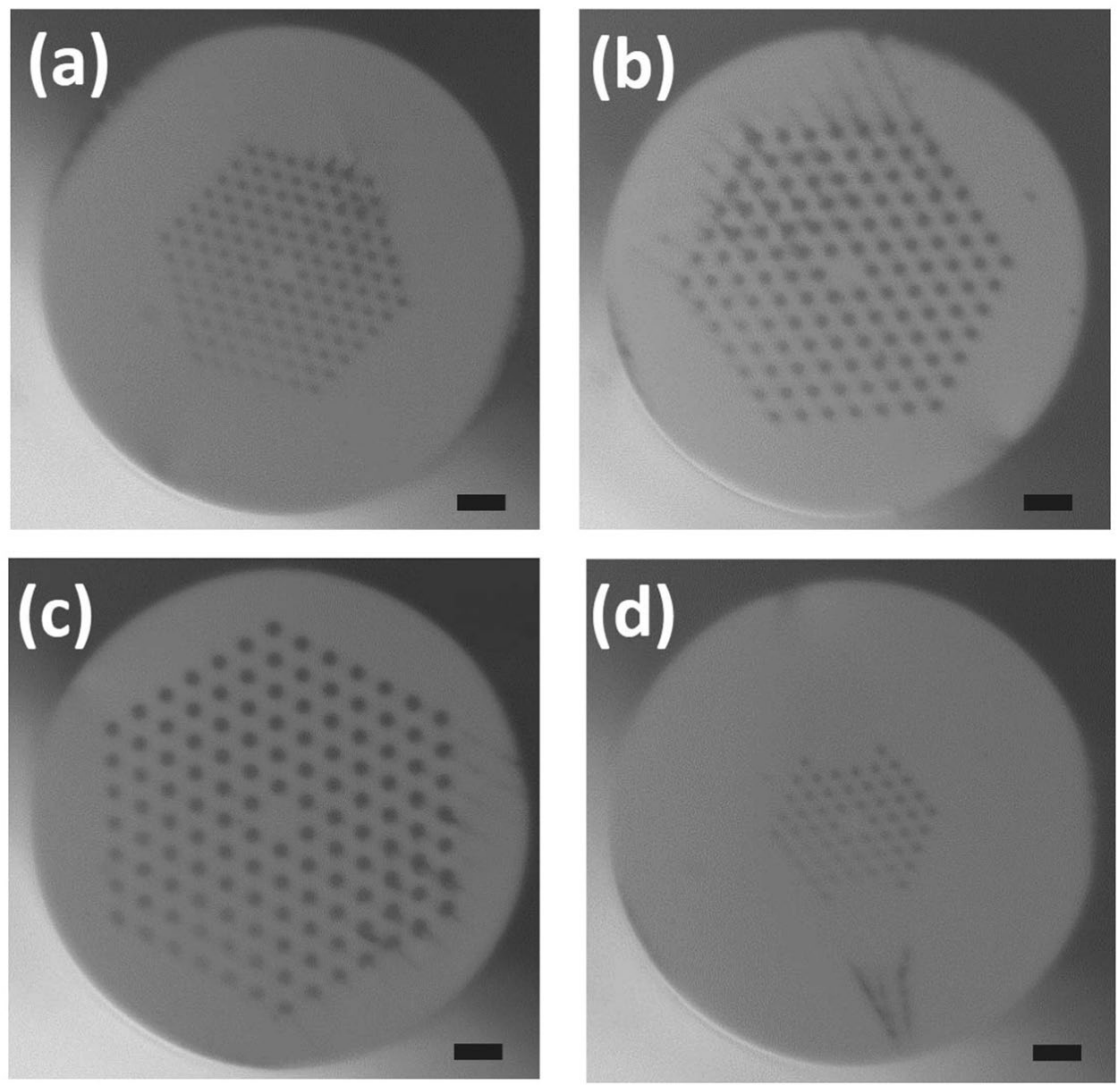
Cross-sections of the LMA PCF: (**a**) LMA-15, (**b**) LMA-20, (**c**) LMA-25, and (**d**) LMA-PM-15. The scale bars in all figures indicate 20 *μ*m.

Fibers tested here included LMA-15, LMA-20, LMA-25, and LMA-PM-15 (NKT Photonics), all of which showing similar dispersion curves with ZDW close to 1230 nm^44^. The first three fibers are normal LMA PCF, with the core size 15 *μ*m, 20 *μ*m and 25 *μ*m, respectively; the last one is a polarization-maintaining LMA PCF with the core size 15 *μ*m. Their cross-sections after cleaving are shown in Fig. 14, and their parameters are listed in Table 2. More detailed information of these fibers can be found on the manufacturer website^44^. We measured the power and spectrum of the generated SC after the collimation parabolic mirror, but before the long-pass filter. The power was measured by a thermopile power meter (PM10, Coherent, Inc.), and the spectra were acquired by the combination of a visible (BlueWave, Stellarnet, Inc.) and a near infrared (EPP2000, Stellarnet, Inc.) spectrometer modules, both corrected for the spectral response. When testing the LMA-PM-15, we added a half-wave plate in front of the focusing lens before the PCF to control the polarization of the incident light.

**Table 2:** LMA PCF parameters. *λ*_z_ represents the ZDW of the fiber. MFD is the mode-field diameter at 1064 nm, and loss is also the attenuation value measured at 1064 nm.

Fiber	Core size (*μ*m)	MFD (*μ*m)	*λ*_z_(nm)	Bend edge (nm)	Loss (dB/km)
LMA-15	15.1	12.8	1222	500	8
LMA-20	19.9	16.5	1226	700	8
LMA-25	25.0	20.9	1235	900	8
LMA-PM-15	14.8	12.6	1221	500	10

The datasets generated during and/or analyzed during the current study are available from the corresponding author on reasonable request.
